# The Braconid *Syntretomorpha szaboi* Papp Is Posing a Great Threat to the Eastern Honeybee, *Apis cerana*

**DOI:** 10.3390/pathogens13050422

**Published:** 2024-05-17

**Authors:** Yanling Xie, Xiaoling Su, Ruike Wei, Lianfei Cao, Huoqing Zheng

**Affiliations:** 1 College of Animal Sciences, Zhejiang University, Hangzhou 310058, China; shellyxie@zju.edu.cn (Y.X.); wrk0326@zju.edu.cn (R.W.); 2 Jinhua Academy of Agricultural Sciences, Jinhua 321017, China; jhmfyjs@163.com; 3 Institute of Animal Husbandry and Veterinary Science, Zhejiang Academy of Agricultural Sciences, Hangzhou 310021, China; beekeepingcao@163.com

**Keywords:** *Syntretomorpha szaboi* Papp, *Apis cerana*, parasitism, braconid, threat

## Abstract

The expansion of pathogen distribution may result in a new threat to the host. The braconid *Syntretomorpha szaboi* Papp is an obligate parasite that targets *Apis cerana*, the Eastern honeybee, engaging in endoparasitism by ovipositing eggs inside the host bee. Although *S. szaboi* has been documented in India and in various regions across China, its epidemiological data are notably lacking. In this study, we summarized the distribution of *S. szaboi* based on the available literature and described the symptoms of infested honeybee workers. We also investigated the infestation rate in 36 apiaries in Zhejiang Province, China, after a new occurrence of the parasite was reported in these regions in 2020. A rapid increase in infestation rate was found from the year 2021 to 2022, reaching 53.88% at the colony level of the sampled colonies in the Jinhua and Wenzhou apiaries. The infestation rate at an individual level in positive colonies reached an average of 26%. A monthly survey showed high seasonal variation in *S. szaboi* infestation, with the peak occurring from May to August. These results suggest that *S. szaboi* poses a great threat to *A. cerana*. Further research is needed to elucidate its epidemiology and pathology and to develop disease prevention and control strategies.

## 1. Introduction

The Eastern honeybee, *Apis cerana*, plays a vital role in local ecosystems and agricultural systems throughout Asia as a native species. However, compared to its globally distributed sister species, the Western honeybee, *Apis mellifera*, there is limited research on the health status of *A. cerana*. This knowledge gap raises concerns for global honeybee health, particularly when considering the potential pathogen spillover events between these closely related sympatric species. The expansion of *Varroa destructor*, an ectoparasitic mite, from *A. cerana* to *A. mellifera* in the 1950s, serves as a stark example of the catastrophic consequences that such spillover events can entail [1].

**Figure 1:**
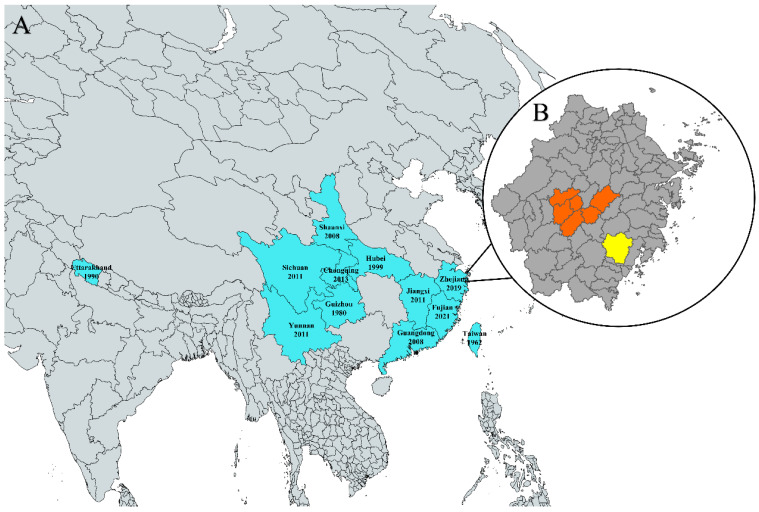
Geographical distribution of *S. szaboi* Papp. (**A**) The blue areas indicate the documented distribution range of the parasite. The years indicate when the parasites were first reported in the areas. (**B**) The orange areas indicate Jinhua, and the yellow area indicates Wenzhou, where sampling was conducted in our study.

Among the factors that negatively affect honeybee health, braconids that parasitize honeybees have received little attention. *Syntretomorpha szaboi* Papp belongs to Ichneumonoidea, Braconidae, Euphorinae, *Syntretomorpha* [2,3]. A male specimen of *S. szaboi* was first described by Papp in Taiwan (1962), but at that time, no host was originally identified [2]. The host was only identified in 1990 when Walker described the adults and larvae parasitizing *A. cerana* in India [3]. However, records of *S. szaboi* have been scarce during the last five decades. The observation of a braconid parasitizing *A. cerana* in China can be traced back to 1966 [4]. No record was reported in the following decades until 1999 when it was recorded in Hubei Province and identified as *S. szaboi* [5]. More records were reported in other provinces in 2008 and afterwards [6,7]. More recently, the presence of *S. szaboi* in *A. cerana* colonies was reported by beekeepers in Zhejiang Province in the eastern China in 2019. In summary, the geographical distribution of *S. szaboi* appears to be large in Asia, ranging from Uttarakhand of India in the west to Taiwan in the east (Figure 1A). However, scientific investigations of this parasite are very limited, and its distribution may extend beyond these documented areas.

*S. szaboi* typically lay their eggs near the honey sac or midgut of their host honeybees, depositing only one egg per host [8]. The larval development of *S. szaboi* occurs within the abdomen of the honeybee. Upon reaching maturity, *S. szaboi* larvae perforate the host’s anus to exit. Subsequently, these larvae commonly construct cocoons in crevices or shaded areas at the bottom of the beehive or in soil, metamorphosing into adults. Infestation by *S. szaboi* results in the death of host honeybees, diminished colony vitality, reduced foraging activity, and a substantial decline in colony population [8]. Considering its widespread and severe impact on honeybees both at the individual and colony levels, *S. szaboi* is a parasite of honeybees that deserves great attention.

To elucidate the epidemiology of this emerging honeybee pathogen, we investigated its prevalence and 1-year seasonal variations using microscopy and PCR detection techniques. We also described the symptoms of honeybee workers infected by this parasite.

## 2. Materials and Methods

### 2.1. Sample Collection

According to beekeepers’ reports, *S. szaboi* infestations were most frequently observed from late June to July. Thus, in July 2021, we sampled 5–10 colonies from each of the 14 apiaries in Jinhua, one of the major beekeeping areas in Zhejiang Province (Figure 1B). Similarly, in July 2022, we collected samples from 3–5 colonies from each of the 15 apiaries within the same area. After the first report in 2021 from a beekeeper in Wenzhou, another major beekeeping area in Zhejiang Province (Figure 1B), we sampled 5 colonies from each of 2 apiaries in 2022 and expanded this to include 3 additional apiaries by the year 2023.

In one of the apiaries that tested positive for *S. szaboi* in Jinhua, a total of 10 colonies were sampled monthly from March 2023 to February 2024 to investigate the seasonal variation in prevalence. For sampling, approximately 100 honeybee workers were randomly sampled from the sideboard of each hive. 

In order to determine the infestation rate at the individual level, the infestation status of 30 worker bees in five positive colonies was determined individually. In addition, honeybee workers near the hive entrance that were suspected of *S. szaboi* infestation were sampled for detection. The collected samples were transported to the laboratory on ice and stored at −80 °C until subsequent analysis.

### 2.2. Dissection and Microscopy Detection

For samples collected in 2021, 30 bees were randomly selected from each colony, and their abdomens were individually dissected to fully expose the abdominal contents under a stereo microscope (Olympus-SZ61, Tokyo, Japan) ×40 magnification (maximum ×45 magnification) to visually determine the presence of *S. szaboi* larvae. The entire dissection procedure was carried out carefully to ensure the integrity of the tissues. 

### 2.3. DNA Extraction 

For colony level detection, the abdomens of 50 bees from each colony were ground to powder with liquid nitrogen, and 200 mg was used for DNA extraction. For individual-level detection, the abdomen of each bee was ground. Total genomic DNA was extracted using the DNA extraction kit following the procedure suggested by the manufacturer (Tiangen, Beijing, China) and resuspended in 50 μL buffer TE. 

### 2.4. PCR Detection and Phylogenetic Analysis 

PCR amplification was performed using primers targeting conserved regions of the parasitoid wasp 28S rDNA gene (28-P1: 5′-CCTGAGAAACCCAAAAG-3′; 28-P2: 5′-CCCAAAGCAGTAGCATCG-3′) [9]. The amplification process was performed under the following cycling conditions: initial denaturation at 94 °C for 2 min; followed by 35 cycles of denaturation at 94 °C for 30 s, annealing at 56 °C for 30 s, extension at 72 °C for 30 s; final extension step at 72 °C for 5 min; and storage at 4 °C. Negative controls (buffer TE instead of sample DNA) were processed simultaneously to identify potential contamination.

The electrophoretic bands of the expected amplicons (about 400 bp) were cut, and the DNA was purified using a SanPrep Column PCR Product Purification Kit (Sangon Biotech, Shanghai, China). The purified amplicons were sent to Sangon Biotech (Shanghai, China) for sequencing. The resulting sequences were compared with those of other Braconidae insects available in GenBank using the Blastn search algorithm. Based on the identification of sequences with high similarity, a phylogenetic evolutionary tree was constructed using MEGA 6 (Arizona State University, Tempe, AZ, USA) with the neighbor-joining method with 1000 bootstraps. The sequences obtained were deposited in GenBank under the accession numbers PRJNA1089366.

## 3. Results

### 3.1. Symptoms

**Figure 2:**
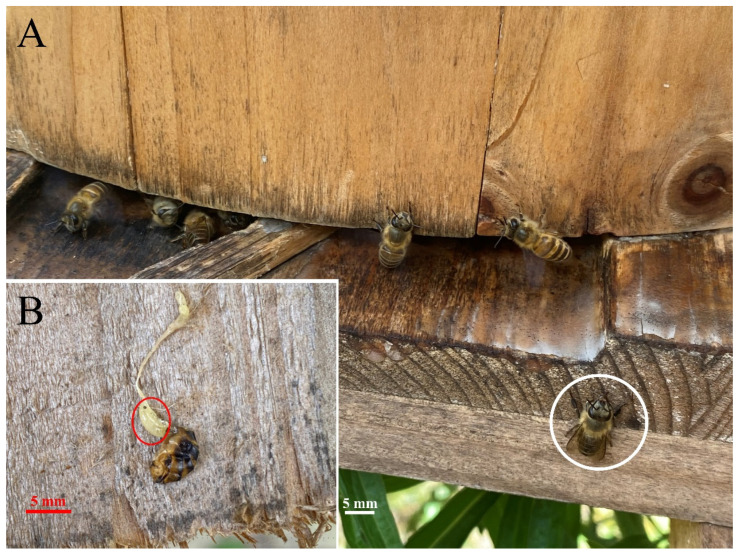
The infected honeybee worker at the entrance of the hive. (**A**) An immobile infested honeybee worker near the entrance of a beehive (in white circle); (**B**) a larva of *S. szaboi* parasitizing in the abdominal cavity can be obviously seen after pulling out the bee’s gut (in red circle).

**Figure 3:**
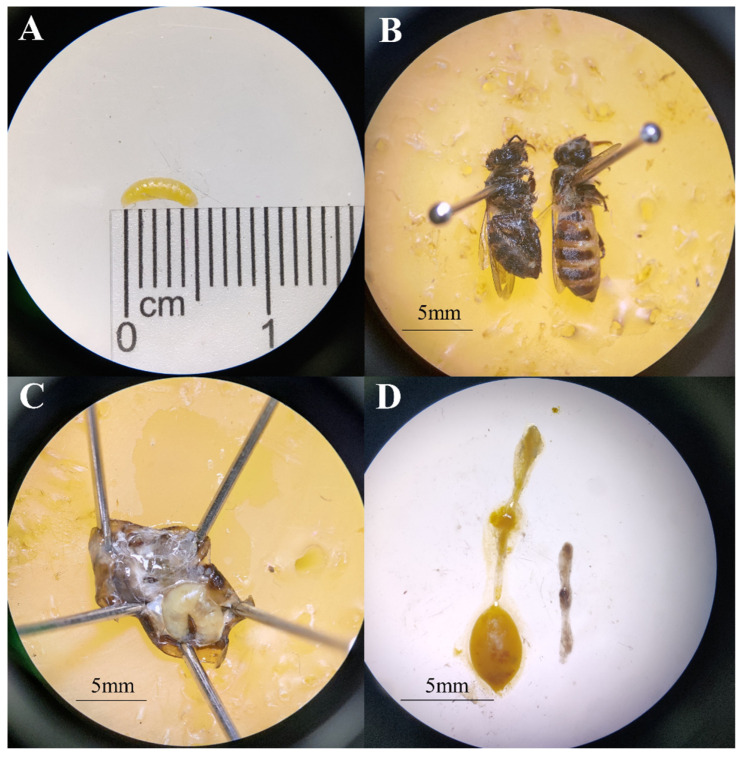
Symptoms of *A. cerana* workers parasitized by *S. szaboi.* (**A**) *S. szaboi* larvae; (**B**) the appearance of a parasitized *Apis cerana* worker (**left**) compared with that of an unparasitized *A. cerana* worker (**right**); (**C**) a dissected abdomen of a worker honeybee parasitized by *S. szaboi*; (**D**) comparison of the intestines of a parasitized bee (**right**) and a normal bee (**left**).

Of the 3000 honeybee workers (30 workers from each of 100 colonies) collected from the inside of hives in 2021, only 1 was found positive by microscopic dissection. But of the 15 suspected workers collected near the hive entrance (Figure 2A), 13 were identified with one larva parasitizing in the abdominal cavity of the bees. Larva can be easily seen by naked eyes when the abdomens of infested workers were pulled off (Figure 2B). These larvae measured an average length of 5.17 ± 1.21 mm (Figure 3A). Their host bees showed reduced activity and a darker body color compared to healthy worker bees (Figure 3B). With the almost complete occupation of the abdominal cavity by *S. szaboi* larva (Figure 3C), the gastrointestinal tract of the infested worker bee appeared fragile and contained minimal contents (Figure 3D).

### 3.2. Infestation Rate Detected with PCR Method

By PCR detection, colonies from 4 of the 14 apiaries were found positive for *S. szaboi*, with an average infestation rate of 4.29% at the colony level in Jinhua in 2021. The infestation rate in all the sampled apiaries showed a rapid increase in 2022, with an average infestation rate of 53.88% at the colony level (Table 1). All the 10 colonies sampled in Wenzhou were positive for *S. szaboi* in 2022, while only 12 of the 25 colonies were positive in 2023. The infestation rate at the individual level in the five positive colonies ranged between 3% and 50%, with an average of 26% (Table 2).

**Figure 4:**
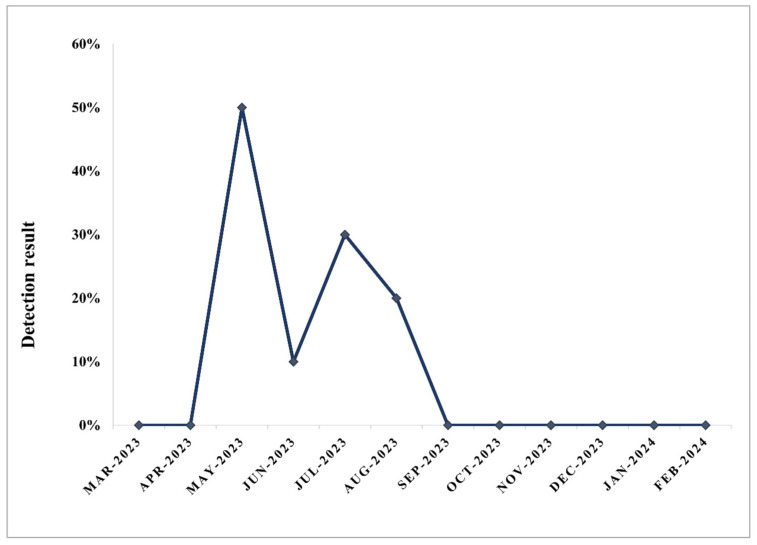
Seasonal variation in infestation rate of *S. szaboi* at colony level in a 1-year sampling period.

The monthly sampling of 10 colonies showed a high seasonal variation in the infestation rate. During the 1-year sampling period, *S. szaboi* was present from May to August, with the highest infestation rate of 50% at the colony level in May, followed by 30% in August (Figure 4).

**Figure 5:**
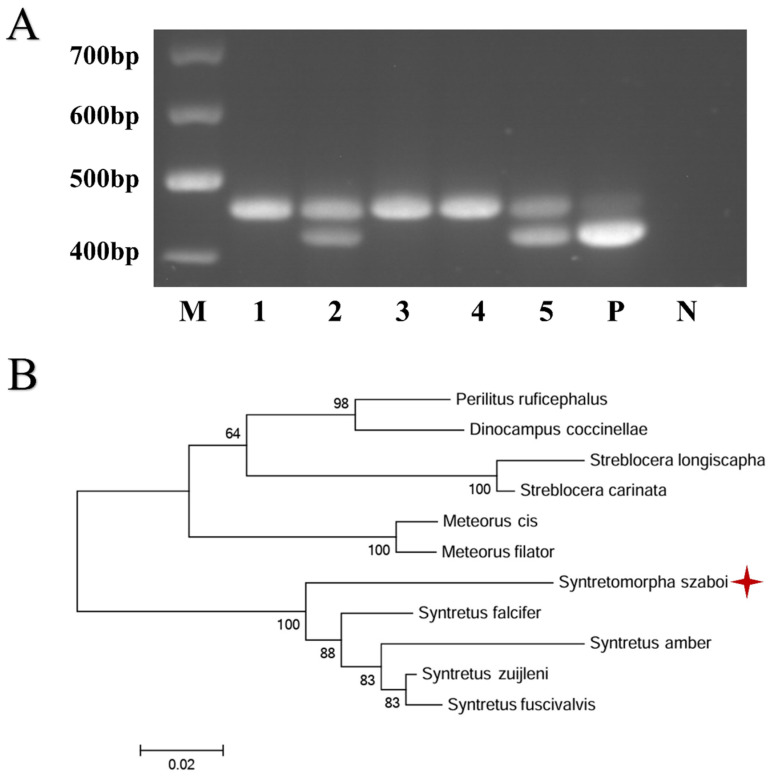
Electrophoresis analysis and phylogenetic analysis of *S. szaboi* (with a red star) amplicons. (**A**) PCR result; (**B**) Evolutionary tree; M: Marker; 1, 3, 4 negative sample showing honeybee amplicons; 2 and 5 positive sample. P: Positive control; N: Negative control.

PCR amplification yielded an amplicon of about 400 bp from *S. szaboi*, showing an extra band on electrophoresis besides the application from honeybee DNA (Figure 5A). The sequence identity between the obtained *S. szaboi* sequence and those of *Syntretus falcifer*, *S. amber*, *S. zuijleni*, *S. fuscivalvis*, and other insects belonging to the family Braconidae is between 83.84% and 91.80% (Figure 5B). 

## 4. Discussion 

Considering the high potential for host switch of pathogens between honeybee species, the survey of pathogen prevalence in *A. cerana* is very important for the global beekeeping industry [10].

The braconid *S. szaboi* did not gain much attention in either the beekeeping industry or the honeybee research field in the past five decades since its first record in 1962 [2]. This is largely due to its limited distribution before 2008. The conspicuous morphological characteristics of both adult and final-instar larvae of *S. szaboi* allow for direct visual observation in beekeeping practice. New reports of its presence by beekeepers in an area normally reflect the expansion of its distribution. Systematic surveys of the geographical distribution of *S. szaboi* are currently lacking. However, a review of the available Chinese literatures clearly indicates an expanding distribution of this endoparasitoid in China [2,3,4,5,6,7,8]. The expansion of *S. szaboi* in China possibly resulted from a significant increase in the number of managed *A. cerana* colonies and the large-scale transportation of colonies. A rapid increase in the infestation rate at the colony level from 2021 to 2022 in Jinhua, Zhejiang Province, suggests a rapid transmission of the parasite after the new invasion in an area. The comparison of the results between microscopic and PCR detection showed that even with the help of a stereo microscope, the infestation status can be severely underestimated. Methods based on PCR detection should be used instead of microscopic detection in future surveys. 

The larvae of parasitoid wasps feed on the tissues or hemolymph of their hosts and develop in their host’s abdominal cavity [11,12,13], altering the food consumption and immunity of the host [11,14,15,16,17]. The impacts that *S. szaboi* infestation causes to honeybees have been largely unexplored. But, the fact that the infestation rate at the individual level in one colony reaches as high as 50% raises serious concerns. As illustrated, the final-instar larvae occupy almost the whole abdominal cavity of their hosts. One can imagine the great damage caused by the parasitism of *S. szaboi* larva to individual honeybee workers. For example, besides the direct impact caused by the feeding in honeybee worker’s abdominal cavity, the digestive function of the host may be severely affected as fragile gastrointestinal tracts were observed in the infested bees in our study. *S. szaboi* can significantly reduce the honeybee population in a colony and decrease their foraging activity, ultimately leading to the colony’s decline or collapse. Additionally, the parasite may transmit and vector honeybee viruses as *V. destructor* does [18,19].

Although the sample size for the seasonal survey was limited and the duration only lasted 1 year in this study, both the field observations and our results suggest a significant seasonal variation in the infestation. Temperature is a key factor affecting the susceptibility of hosts and the virulence of parasites [20]. When discussing the relationship between parasitic wasps, their hosts, and temperature, the existing literature largely focuses on the adaptability of parasitic wasps to around 25 °C and their intolerance to high temperatures (≥30 °C) [21,22]. However, in our study, it was found that *S. szaboi* are more prevalent in late spring and summer when the temperature is relatively higher (25–40 °C), suggesting their unique physiological and behavioral adaptation strategies to high temperatures. For example, *S. szaboi* may cope with high-temperature stress by enhancing its physiological capabilities or actively seeking cooler environments. This adaptability may be crucial for the ecological dynamics of parasitic wasp populations.

No record of the infestation of *S. szaboi* in *A. mellifera* has been reported yet. Although the parasitic relationship between parasite and host depends on their complex co-evolutionary dynamics, certain conditions can induce a parasite to expand its host range to a closely related species from its original host. China manages approximately 5 million colonies of *A. cerana* and *A. mellifera* each, resulting in a remarkably high density of these sympatric honeybee species, significantly increasing the likelihood of host switching for honeybee pathogens [23]. Consequently, the spread of *S. szaboi* underscores an escalating global risk to honeybees. More research is needed to elucidate the biology, epidemiology, and pathogenicity of the parasite before it causes disastrous damage to honeybees across the world. 

## 5. Conclusions

*S. szaboi* poses great threats to *A. cerana* with a high infestation rate both at the colony level and the individual level. The infestation rate of *S. szaboi* varies significantly between seasons, showing an adaptability of the parasite to relatively high temperatures. Given the high potential for pathogens to switch hosts between honeybee species, it is crucial to monitor the prevalence of *S. szaboi* to protect the global beekeeping industry.

## Figures and Tables

**Table 1:** Infestation rate of *S. szaboi* at colony level in apiaries sampled from 2021–2023 determined with PCR detection.

2021		2022		2023	
Apiary	Infestation Rate	Apiary	Infestation Rate	Apiary	Infestation Rate
Jinhua-1	0/5 (0%)	Jinhua-1	0/3 (0%)	Wenzhou-1	5/5 (100%)
Jinhua-2	1/10 (10%)	Jinhua-2	4/5 (80%)	Wenzhou-2	0/5 (0%)
Jinhua-3	0/10 (0%)	Jinhua-3	4/5 (80%)	Wenzhou-3	0/5 (0%)
Jinhua-4	1/5 (20%)	Jinhua-4	3/4 (75%)	Wenzhou-4	5/5 (100%)
Jinhua-5	0/5 (0%)	Jinhua-5	1/5 (20%)	Wenzhou-5	2/5 (40%)
Jinhua-6	0/10 (0%)	Jinhua-6	0/5 (0%)		
Jinhua-7	0/5 (0%)	Jinhua-7	2/5 (40%)		
Jinhua-8	1/10 (10%)	Jinhua-8	2/3 (66%)		
Jinhua-9	0/5 (0%)	Jinhua-9	3/4 (75%)		
Jinhua-10	2/10 (20%)	Jinhua-10	5/5 (100%)		
Jinhua-11	0/10 (0%)	Jinhua-11	2/5 (40%)		
Jinhua-12	0/5 (0%)	Jinhua-12	2/5 (40%)		
Jinhua-13	0/5 (0%)	Jinhua-13	0/5 (0%)		
Jinhua-14	0/5 (0%)	Jinhua-14	0/5 (0%)		
		Jinhua-15	5/5 (100%)		
		Wenzhou-1	5/5 (100%)		
		Wenzhou-2	5/5 (100%)		

**Table 2:** Infestation rate of *S. szaboi* in individual bees in 5 colonies.

Apiary	Colony	Detection Rates
Wenzhou-1	2	15/30 (50%)
	4	11/30 (37%)
	5	1/30 (3%)
Wenzhou-4	1	6/30 (20%)
	2	6/30 (20%)

## Data Availability

Data are contained within this article.
